# Identification of different attitudes towards paratuberculosis control using cluster analysis applied on data from an anonymous survey among German cattle farmers

**DOI:** 10.1186/s13620-021-00204-3

**Published:** 2021-09-15

**Authors:** Veit Zoche-Golob, René Pützschel, Esra Einax, Karsten Donat

**Affiliations:** 1 Thuringian Animal Diseases Fund, Animal Health Service, Jena, Thuringia Germany; 2 Department Biological Safety, German Federal Institute for Risk Assessment, Unit Epidemiology, Zoonoses and Antimicrobial Resistance, Diedersdorfer Weg 1, 12277 Berlin, Germany; 3 Saxon Animal Disease Fund, Animal Health Service, Dresden, Saxony Germany; 4 Clinic for Obstetrics, Gynaecology and Andrology with Veterinary Ambulance, Justus-Liebig-University, Giessen, Hesse Germany

**Keywords:** Questionnaire, Johne’s disease, Motivation, Mindset, Risk perception

## Abstract

**Background:**

Paratuberculosis is a common disease in ruminants, causing economic losses in livestock farming, and a relationship between the exposure to its causative agent *Mycobacterium avium* subsp. *paratuberculosis,* and Crohn’s disease in humans is discussed. Despite this, only a minority of cattle farmers have enroled in voluntary control programmes in most countries. Therefore, this study aimed at investigating the farmer’s opinion on paratuberculosis and their motivations to participate in a control programme. The objective was to identify different groups among farmers regarding their motivation and thereby contribute to a better understanding of farmers’ attitudes towards paratuberculosis control.

**Results:**

Two hundred twenty-five farmers responded to questionnaires that were distributed among cattle farmers in Saxony and Thuringia, federal states of Germany, together with boot-swab sampling sets for a free and anonymous herd-level paratuberculosis test. Among them, dairy herds and large herds were overrepresented. A hierarchical cluster analysis of the farmers’ answers resulted in four groups that we tagged as ‘informed sceptics’, ‘deniers’, ‘affected supporters’ and ‘free supporters’. In all groups, the majority considered paratuberculosis a threat to the public image of cattle farmers. Nearly all participants wanted to know the paratuberculosis herd status of purchased cattle. In contrast to the supporters, the informed sceptics and the deniers did not consider paratuberculosis a dangerous epizootic disease and would not welcome a mandatory control programme. The deniers and the affected supporters, but not the informed sceptics and the free supporters, assumed that their herd is affected by paratuberculosis. Unlike the deniers, all other groups would enrol in a control programme if the pathogen would have been found in their herd. Protecting future profitability and improving animal health were the two most important motivations to control paratuberculosis in all groups followed by aspects related to the marketing of breeding cattle. Most frequently, the costs and the assumed inaccuracy of diagnostics tests were mentioned as obstacles that hamper programme enrolment.

**Conclusions:**

Significantly different attitudes of farmers regarding paratuberculosis control were identified. Therefore, tailored rather than uniform communication strategies are required to enhance participation in voluntary paratuberculosis control programmes.

**Supplementary Information:**

The online version contains supplementary material available at 10.1186/s13620-021-00204-3.

## Background

Paratuberculosis, or Johne’s disease, is a granulomatous enteritis of ruminants including cattle, sheep, goats, and red deer [1] that is caused by *Mycobacterium avium* ssp. *paratuberculosis* (MAP). As an effective therapy is not available, affected cows may suffer from intermittent diarrhoea, oedema and weight loss and finally die [2]. Paratuberculosis is an O.I.E.-listed terrestrial animal disease that occurs worldwide. Based on laboratory test data, estimates for herd-level prevalence in dairy cattle vary between < 1 and > 40% among countries [3]. Relevant economic losses in dairy farming are caused by decreases in milk yield [4], slaughter weight [5], and fertility [6]. Recently, a meta-analysis provided evidence for positive ELISA tests for MAP in Crohn’s disease patients, but due to knowledge gaps in understanding the role of MAP in the development of human disease, the impact of MAP on public health cannot be quantified yet [7]. A zoonotic potential of paratuberculosis, even if hypothetical, could have negative effects on the whole dairy industry [8].

Paratuberculosis control activities and strategies differ widely at present. Formal control programmes are present in some, mainly more highly developed countries with advanced veterinary services. On the other hand, most of the countries in Asia, Africa, South and Central America have no formal control programmes, and in most low-middle income countries any kind of control activities is absent. Participation is completely voluntary in most countries with control programmes, whereas compulsory participation was legislated in Japan, Norway and Lower Saxony, a federal state of Germany. Reporting of MAP suspicion is mandatory in Sweden. Full or partial financial support, assistance or compensation to farmers for one or more operational aspects of the control program is provided in numerous countries [3]. Market access restrictions for non-participation can be an incentive as well. For example, the Dutch dairy industry bans milk delivery from dairy herds that are not involved in the programme, resulting in a participation of nearly all dairy farmers [9].

As most of the control programmes worldwide are voluntary, their success depends on enrolment and retention. It is essential to account for farmers’ motivators and obstacles in order to convince them to enrol in the programmes and to implement recommended measures in their herds [10]. The participation in voluntary control programmes often remains low even though the adverse effects of paratuberculosis on animal health and the benefits of the control programmes have been extensively communicated for decades. For example, in Denmark, only 28% of the dairy herds are part of a producer paid control programme [11].

Attitudes of farmers towards the disease and their beliefs have been found to importantly influence their motivation to implement suggested management improvements in their farms [12]. Farmers’ decision making depends on intrinsic factors like farmers’ attitudes, beliefs and norms and extrinsic circumstances that include farmers’ knowledge about the disease and their ability to deal with it, as well as obstacles like limited time or money [13]. In Alberta, Canada, benefits and low costs for the farmers were extensively communicated, and a reasonable percentage of the dairy farmers (65%) are enroled in a voluntary control programme [14]. To establish high participation rates, herd health veterinarians should be enabled and encouraged to promote the programme as they have been regarded as reliable and trustworthy sources of advice on disease management by farmers [15, 16]. Furthermore, farmers’ intentions to improve herd health in general and their concern about threatening consumer health can be important motivators to enrol in paratuberculosis control programmes [11, 17].

Voluntary control programmes have been in force for one or two decades in several German federal states e.g. in Hesse, Thuringia or Saxony [16, 18]. In these programmes, several diagnostic approaches have been applied, and environmental sampling has been used as a first-line sampling approach to identify MAP positive herds. Boot-swab sampling as described by Eisenberg [19] and evaluated by several studies [20, 21] is widely used because it is an easy-to-use sampling approach that can be done by the farmers themselves. Furthermore, detecting MAP or MAP genome from boot-swabs by culture or by quantitative polymerase chain reaction (qPCR), respectively, provides highly specific information whether a herd is MAP positive by testing only one sample.

Financial incentives like full or partial defrayal of the costs for testing or for veterinary advice, or compensation for culled test-positive animals are provided in most regional programmes in Germany. Despite this, participation is still low reflecting a lack of farmers’ motivation to participate in the programmes.

Studying farmers’ motivations and attitudes towards voluntary paratuberculosis control in Germany may contribute to a better understanding of motivators and barriers to enrolment in paratuberculosis control, thereby helping to improve participation. Therefore, this study aimed at investigating farmers’ attitudes towards paratuberculosis control, and their motivations to participate in an existing voluntary control programme. The objective was to identify different groups among them regarding their motivation and thereby contribute to a better understanding of the farmers’ mindset towards paratuberculosis control.

## Results

### Response rate and demographics of respondents

Two hundred twenty five of the 625 distributed questionnaires were returned together with boot-swab samples, 117 from Thuringia and 108 from Saxony. All answers are available from Additional file 1. Table 1 compares frequency distributions of the respondents’ herds in terms of federal state and herd size with those of the registered cattle holdings in Saxony and Thuringia.

**Table 1:** Frequency distributions of the participating farms (sample) in comparison to the registered cattle holdings in Saxony and Thuringia (population)

	Saxony				Thuringia				Total			
	Sample		Population		Sample		Population		Sample		Population	
Herd size	n	%	n	%	n	%	n	%	n	%	n	%
≤ 10	6	5.6	3561	66.2	4	3.4	2134	67.8	10	4.4	5695	66.8
11–100	27	25	1300	24.2	14	12	637	20.2	41	18.2	1937	22.7
101–500	41	38	406	7.5	56	47.9	301	9.6	97	43.1	707	8.3
> 500	33	30.6	112	2.1	39	33.3	76	2.4	72	32.0	188	2.2
n.a.	1	0.9	–	–	4	3.4	–	–	5	2.2	–	–
Total	108		5379		117		3148		225		8527	

Results from qPCR testing for MAP genome were positive for 67, negative for 151 and could not be evaluated for 7 out of the 255 boot-swab samples. Relative frequencies are given in Table 2. Associations between these test results and herd size or production type as well as a prevalence estimation based on these data for Thuringia and Saxony are published elsewhere [22]. The frequencies of answers to the questions in part 2 to 4 are presented in Table 3.

**Table 2:** Within-group and total frequencies of answers and results of testing boot-swabs for *Mycobacterium avium* ssp. *paratuberculosis* genome

		Group 1	Group 2	Group 3	Group 4	Total
		‘Informed sceptics’	‘Deniers’	‘Affected supporters’	‘Free supporters’	
	Count	44	37	46	98	225
		%	%	%	%	%
State	Saxony	38.6	54.1	50.0	49.0	48.0
	Thuringia	61.4	45.9	50.0	51.0	52.0
Herd type	Dairy cows	70.5	73.0	80.4	62.2	69.0
	Suckler cows	18.2	10.8	8.7	26.5	9.8
	Mixed	9.1	13.5	8.7	9.2	18.7
	Missing answer	2.3	2.7	2.2	2.0	2.2
Herd size ^a^	≤ 10	4.5	2.7	0.0	7.1	4.4
	11–100	15.9	10.8	10.9	25.5	18.2
	101–500	47.7	43.2	32.6	45.9	43.1
	> 500	31.8	35.1	56.5	19.4	32.0
	Missing answer	0.0	8.1	0.0	2.0	2.2
Paratuberculosis has been known for... ^a^	Today	4.5	0.0	2.2	2.0	1.3
	The recent year	6.8	2.7	0.0	13.3	7.6
	≤ five years	29.5	21.6	13.0	30.6	25.5
	> five years	59.1	67.6	84.8	54.1	63.6
	Missing answer	0.0	8.1	0.0	0.0	2.2
Enrolment^a^	Yes	2.3	18.9	54.3	15.3	32.2
	No	88.6	62.2	37.0	73.5	67.7
	Missing answer	9.1	18.9	8.7	11.2	11.6
Boot-swab result ^a^	MAP-positive	15.9	51.4	52.2	17.3	3.1
	MAP-negative	79.5	43.2	47.8	79.6	29.8
	Not evaluable	4.5	5.4	0.0	3.1	67.1

^a^ The frequencies were not independent from the groups in a Bayesian Poisson regression model for contingency tables

**Table 3:** Relative frequencies [%] of the of the participants’ answers (Part 2–4) and median ranks on a scale from 1 (agreement) to 5 (disagreement) for the identified groups of cattle farmers

Question	Frequencies of answers					Mean rank			
Answer	Yes	Rather yes	Missing answer	Rather no	No	Group 1	Group 2	Group 3	Group 4
Rank	1	2	3	4	5	‘Informed sceptics’	‘Deniers’	‘Affected supporters’	‘Free supporters’
Do you consider paratuberculosis a dangerous epizootic disease?	36.9	36.0	2.2	20.9	4.0	4	3	1	2
Do you think that paratuberculosis threatens your cattle business’ profitability?	48.0	28.9	1.8	19.6	1.8	2	2	1	1
Do you fear an impact of MAP exposure on your personal health by its believed potential of causing Morbus Crohn?	28.0	25.8	3.1	36.0	7.1	2	4	2	2
Do you fear that a potential public health impact of MAP exposure will damage the image of the dairy and beef sectors?	66.2	22.2	2.2	6.7	2.7	1	2	1	1
Do you assume or know that your herd is affected by paratuberculosis?	20.4	15.1	2.2	34.7	27.6	4	2	1	4
Will the detection of MAP in your herd prod you into enroling in a paratuberculosis control programme?	51.1	35.6	4.0	7.6	1.8	2	4	1	1
Do you want to know the paratuberculosis status of the herd of origin when buying breeding cattle?	87.6	10.7	1.3	0.4	0.0	1	1	1	1
Would you welcome a mandatory control programme in Germany?	37.3	36.9	3.1	15.6	7.1	4	3	1	1
If you are / were participating in a control programme, what will / would be your motivation?									
to protect future profitability	74.7	16.9	6.2	0.9	1.3	1	2	1	1
to ease the marketing of breeding cattle	60.9	18.2	5.8	9.3	5.8	1	2	1	1
to protect the own healthiness	50.7	27.1	6.2	12.9	3.1	2	3	1	1
to improve general animal health	73.8	20.0	4.9	1.3	0.0	1	2	1	1
because I perceive paratuberculosis a threat to food safety	25.8	22.2	7.6	33.3	11.1	4	4	2	2
because I don’t want to miss the boat	34.2	24.0	8.4	21.3	12.0	2	3	2	2
financial incentives of the Animal Disease Fund	58.2	23.6	7.1	7.6	3.6	1	2	1	1
enrolment of my business partners or peers	17.8	21.3	12.4	24.9	23.6	4	3	3	3
If you are / were not participating in a control programme, what will / would be your motivation?									
because the programme is not mandatory	12.9	14.7	48.0	11.1	13.3	3	3	3	3
the costs of the diagnostic tests	25.8	14.7	46.2	8.9	4.4	2	2	3	3
the additional efforts to improve hygiene	16.9	10.7	48.0	13.3	11.1	3	2	3	3
because I cannot cull positive cows	10.7	13.8	48.0	13.8	13.8	3	3	3	3
because I think the diagnostic tests are of low accuracy	14.2	13.8	47.6	14.2	10.2	3	2	3	3
because I do not recognise a threat to food safety	8.9	13.3	47.6	15.6	14.7	3	3	3	3

### Identified groups of cattle farmers

**Fig. 1:**
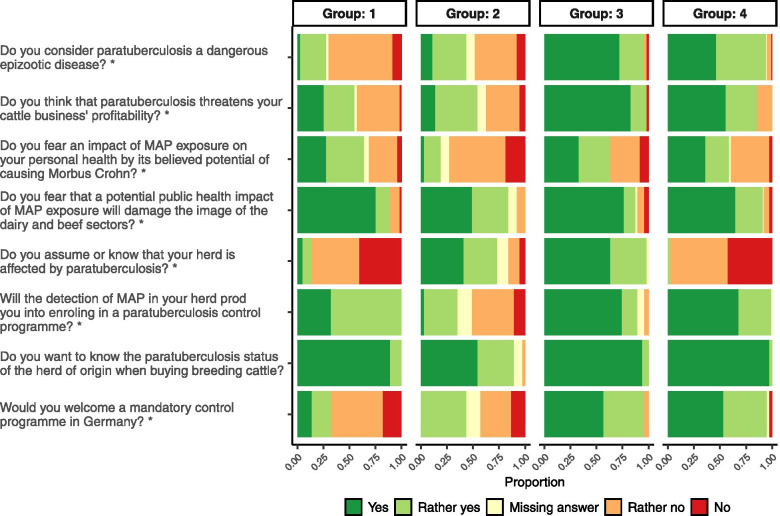
Relative frequencies of answers to questions in part 2 (attitudes, opinions, awareness) within groups. The proportions of answers to the questions marked with an asterisk (*) were not independent from the groups in a Bayesian Poisson regression model for contingency tables

Four groups of cattle farmers were identified by cluster analysis (Fig. 1) assigning each farmer to one group as given in Additional file 1. The groups consisted of 44 (Group 1), 37 (Group 2), 46 (Group 3) and 98 (Group 4) farmers out of 225 participants. At four clusters, the average distance within clusters and the separation index were 0.20 and 0.063, respectively. The bootstrap resampling yielded Jaccard similarity values for the four clusters of 0.55, 0.51, 0.56, and 0.44.

**Fig. 2:**
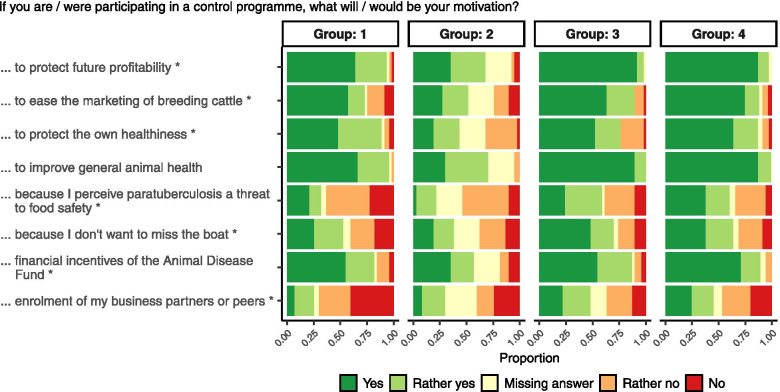
Relative frequencies of answers to questions in part 3 (motivations) within groups. The proportions of answers to the questions marked with an asterisk (*) were not independent from the groups in a Bayesian Poisson regression model for contingency tables

**Fig. 3:**
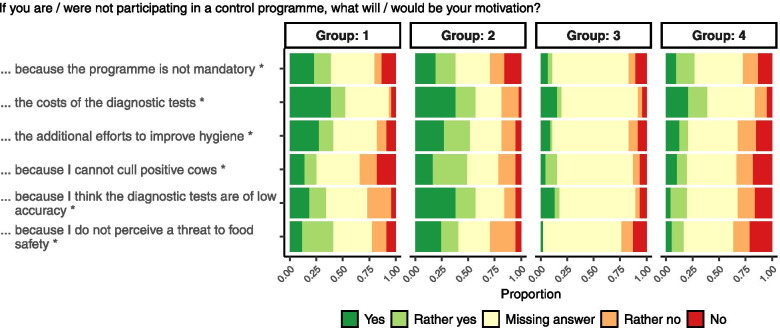
Relative frequencies of answers to questions in part 4 (obstacles) within groups. The proportions of answers to the questions marked with an asterisk (*) were not independent from the groups in a Bayesian Poisson regression model for contingency tables

The answers were ranked from 1 (agreement) to 5 (disagreement). Groups 1 and 2 differed from groups 3 and 4 by answering the questions if paratuberculosis was considered a dangerous disease and if a mandatory control programme would be welcomed (Tables 3 and 4). Groups 1 and 4 were distinguished from groups 2 and 3, respectively, by believing that their herd was not affected by paratuberculosis. In contrast to farmers of group 1, those of group 2 would not participate in a control programme if their herd would have been identified as positive. The majority of group 3 farmers had already joined the programme (54.3%), whereas only 15.3% of group 4 farmers had already decided to participate in the programme at the time of the survey. A simplified description of the groups resulting from the cluster analysis is summarized in Table 4. The differences between the groups in the answers to the questionnaire are presented in Table 3 and Figs. 1,2 and 3.

**Table 4:** Simplified schematic presentation of groups resulting from cluster analysis

Clustering characteristics		Do you assume or know that your herd is affected by paratuberculosis?		Additional characteristics
		No	Yes	
Do you consider paratuberculosis a dangerous epizootic disease? Would you welcome a mandatory control programme in Germany? Do you think that paratuberculosis threatens your cattle business’ profitability?	No	Group 1 **‘Informed Sceptics’** Participation^a^: Yes	Group 2 **‘Denier**s**’** Participation^a^: No	**All Groups:** -want to know the paratuberculosis status of the herd of origin when buying breeding cattle - fear that a potential public health impact of MAP exposure will damage the image of the dairy and beef sector
	Yes	Group 4 **‘Free Supporters’** Participation^a^: Yes	Group 3 **‘Affected supporters’** Participation^a^: Yes	

^a^Answer to the question ‘Will the detection of MAP in your herd prod you into enroling in a paratuberculosis control programme?’

### Motivations for enrolment in a Paratuberculosis control Programme

For farmers of all groups, expected improvement of animal health in general, protection of future profitability of their business and financial incentives were the main reasons for enrolment in a control programme. Expectation of better market access for breeding cattle and the protection of their own health were relevant motivations for the majority of farmer in all groups as well. Only farmers in groups 3 and 4 were motivated by perceiving paratuberculosis a threat to food safety. In this study, potential enrolment of their peers or business partners in a control programme was not a relevant motivation of farmers of all groups to participate as well. Figure 2 shows the proportions of the answers within groups to the questions in part 3 of the questionnaire dealing with motivations to take part in a control programme.

### Obstacles hampering participation in cases of non-enrolment

Most frequently, the costs and the assumed low accuracy of diagnostics tests were mentioned as obstacles that hamper participation, followed by the expected additional efforts to improve hygiene management. In contrast to other groups, a relevant proportion of group 2 farmers did not perceive paratuberculosis a threat to food safety. Missing capacities to cull MAP-positive cows were rarely mentioned as obstacle for participation. The non-mandatory character of the programme was relevant to only a minority of farmers, mainly to those of groups 1 and 2.

Farmers who were identified as supporters of paratuberculosis controls (groups 3 and 4) frequently did not answer questions dealing with obstacles that hamper participation in a control programme, resulting in a response rate of only 53.8% to questions of part 4. The proportions of the answers within groups to the questions of part 4 are presented in Fig. 3.

## Discussion

The present study aimed at investigating the mindset of farmers toward paratuberculosis control in a voluntary setting and to identify different groups among them regarding their attitudes and key motivations. In this study, a cluster analysis including answers to eight questions identified four groups of cattle farmers that are described in detail below. Additional demographic data demonstrated that 67.1% of the respondents did not enrol in a voluntary paratuberculosis control programme, even though they had known about paratuberculosis for more than 5 years. This reflects that knowledge transfer does not sufficiently motivate control programme enrolment and underlines the need to evaluate the motivations and obstacles for farmers’ participation.

Farmers are not a homogenous group and their behaviour is influenced by their attitudes and beliefs [23]. Previous studies have shown, that farmers’ decision making depends on intrinsic factors like farmers’ attitudes, beliefs and norms and extrinsic circumstances that include farmers’ knowledge about the disease and their ability to deal with it. Limited time or money may be obstacles. Furthermore, farmer’s decisions are modified by social-psychological influences like problem awareness, perception of responsibility, effectiveness of recommended strategies, farmers’ ability to implement recommended management practices and perceived benefits and disadvantages [24–26].

### Interpretation of the identified clusters

In a cluster analysis, it is a major challenge to interpret the resulting clusters. The algorithm does not provide any borders or rules to identify the clusters but only assigns every observation to a cluster. The interpretation has to rely on the different distributions of the variable values between clusters.

Based on their beliefs in being affected by paratuberculosis, their willingness to enrol in the programme and their opinion on the degree to which paratuberculosis is hazardous, we designated the clusters as ‘informed sceptics’ (group 1), ‘deniers’ (group 2), ‘affected supporters’ (group 3) and ‘free supporters’ (group 4). A Canadian study based on semi-structured interviews of 25 dairy farmers in Alberta identified four groups of farmers (proactivists, disillusionists, unconcerned, deniers) concerning their beliefs in the importance of the disease and in recommended control strategies [27]. In their study, farm economics, animal health and welfare were reasons for farmers to consider paratuberculosis of increased importance, whereas the potential link to Crohn’s disease was relevant only for some farmers. Both outcomes are in line with our results, although the Canadian researchers identified the groups of farmers by a completely different method than the quantitative cluster analysis used in our study. They used grounded theory to form the groups of farmers in a qualitative way [27].

The ‘proactivists’ of the Canadian study [27] are similar to our supporters. They acknowledge their responsibility for food safety and the image of the dairy industry and would participate in a control programme if the pathogen was detected in their herd. In our study, it is important to differentiate between free and affected supporters instead of joining them as ‘proactivists’ because our study was performed about two decades after the first voluntary control programme came into force in Saxony and Thuringia. Therefore, a relevant proportion of herds had been identified as MAP-positive several years before this survey was conducted. Affected supporters may have experienced a noticeable improvement of animal health in general, and free supporters may even have gained a certificate indicating likely herd freedom from paratuberculosis. A proactive attitude of the farmer would have led to programme enrolment in both cases: because the herd is affected, or in order to gain a certificate. Like the Canadian authors [27], we identified a group of ‘deniers’ in our study. They do not consider paratuberculosis a dangerous disease and do not recognise the importance of paratuberculosis prevention and control, and therefore, would not take part in the programme even if their herd was affected. Characteristics of the Canadian ‘disillusionists’ and ‘unconcerned’ [27] were found among our ‘informed sceptics’. They were sceptical regarding the benefit of the control measures, e. g. the diagnostic tests and the management practices, and did not perceive paratuberculosis a threat to food safety. On the other hand, they were interested in available research [27]. Our cluster analysis did not result in a differentiation of this group.

Cluster analysis had been used in former studies on the motivation of dairy farmers to improve mastitis management [28]. Despite differing methodological approaches, our analysis provided similar results as studies using regression analysis [29] or qualitative methods adopted from social sciences [27]. Interestingly, the Canadian study [27] and ours each identified comparable groups. However, great caution is required for generalisation of these grouping schemes. For farmers of all groups, the expected improvement of animal health in general, the future profitability of their business and financial incentives were the main motivators for participation in a control programme. This is in line with the results of another Canadian study performed in 224 dairy herds where improving herd health and increasing net profit and longevity were identified as the top three goals among participants and nonparticipants [29]. Several studies support the hypothesis that improving animal welfare is an important motivation for farmers to control infectious diseases, mastitis or lameness [15, 28]. Regarding paratuberculosis control, about 40% of dairy farmers assumed that calf and herd health would improve after implementation of paratuberculosis control measures [30]. Overall, the farmers of our study were concerned about the public image of the dairy and beef industry with respect to a potential public health impact of MAP exposure. The participation of their peers and business partners and, surprisingly, food safety, were rated less important in all groups. A relevant proportion of the deniers and to a less extent of the informed sceptics did not see any threat to food safety coming from paratuberculosis. Based on their view that paratuberculosis was not a dangerous epizootic disease and in contrast to the other groups, the informed sceptics and the deniers would not welcome a mandatory control programme. Their main obstacles hampering participation were the expected diagnostic costs and expected additional efforts to improve hygiene. Obviously, the deniers were not concerned about paratuberculosis. In contrast to the other groups, a majority of them did not fear that MAP exposure impacted their personal health. Interestingly, an important reason for the informed sceptics and the deniers not participating in the program was its voluntary nature. In particular, the deniers thought that the current diagnostic test for paratuberculosis lacked accuracy. This was less relevant to the other groups. In contrast, neither of the supporter groups were deterred by the diagnostic costs and the efforts that may be required to improve hygiene. Both groups of supporters stated that they would participate in a voluntary control programme if paratuberculosis would have been detected in their herd, and would welcome a mandatory programme. It can be assumed that a part of the affected supporters – a proportion of 54.3% of this group had already been enroled in the programme – had experienced economic benefits from their efforts to control the disease. The affected supporters clearly considered paratuberculosis a dangerous epizootic disease. Lacking capacity to cull MAP-positive cows was not mentioned as a main obstacle, neither by the deniers nor the supporting groups. When buying breeding cattle, the herd status of origin was important for all responders except for very few deniers. Among all groups, but to a lesser extend among the deniers, farmers expected better market access for breeding cattle if they controlled the disease in their herd.

In the group of the deniers, as many MAP-positive boot-swabs (51.4%) were detected as in the group of the affected supporters. Despite a comparable probability to be affected, both groups differed markedly in their beliefs in the effectiveness of paratuberculosis control and its relevance for the own business.

A high proportion of participants and nearly all supporters (groups 3 and 4) answered the questions in part 3 of the questionnaire focussing on the motivation to participate in the programme. Nearly a quarter of the deniers (group 2) did not answer these questions, probably because they had decided not to enrol in the programme, and the questions with respect to motivations to participate were out of their scope (Fig. 2). Only a minority of farmers who were motivated to participate in a control programme, paid attention to the questions in part 4 of the questionnaire asking for obstacles hampering participation. The proportion differed notably between the identified groups (Fig. 3). This may explain the high proportion of missing answers to questions in part 4.

### Limitations of the study

Despite its anonymous character, our study was not based on random sampling as a formal randomisation process was not included in the study design. Instead, the questionnaires were distributed together with sampling sets for boot-swabs at extension courses for farmers. This convenience sampling resulted in a biased selection of cattle holders. Owners or managers of large herds and of dairy herds were overrepresented: Farmers with more than 100 cattle represented three-fourth of the respondents but only a tenth part of all cattle holders in Saxony and Thuringia (Table 1). On the one hand, this overrepresentation of farmers with large herds may have reflected their greater interest in further education. In both federal states of the study, most employed managers of large co-operative farms are graduates from university or technical college, or hold at least a master craftsman’s diploma. Taking into account these circumstances, an above-average knowledge level of the responders can be assumed. A higher level of self-assessed knowledge about the disease was positively associated with participation in the control programme in a Canadian study [29]. On the other hand, in large herds the impact of paratuberculosis is presumably greater than in smaller herds, as herd size is positively associated with MAP detection at herd level [14, 22]. Furthermore, management practices that are known to increase the spread of MAP within a herd are more common in larger herds (e. g. large calving areas, multiple cows in one calving pen, or feeding colostrum from untested cows to multiple calves). These management practices as well as the frequent trading of animals are less common in small herds. Therefore, the results of our study provide valuable indications of how to deal with different groups among owners and managers of large herds, even though they may not be generalised to all cattle farmers.

Another potential source of bias was the overrepresentation of dairy farmers. We decided to include both farm types in the cluster analysis and not to analyse them separately, because a similar proportion of both production types was enroled in the paratuberculosis control programmes in both federal states. The aim of our cluster analysis was to identify clusters among the participants solely based on their attitudes towards paratuberculosis and its control, not on the production type of their herds. We are aware that paratuberculosis is generally regarded as a problem of the dairy industry more than of the beef industry. Nonetheless, beef herds are affected by paratuberculosis as well. Therefore, we combined data from beef and dairy herds in our analysis. Our study did not aim at identifying differences between beef and dairy farmers in their attitudes and opinions regarding paratuberculosis and its control. The question as to whether these attitudes depend on production type is left to further research.

The mean Jaccard similarity value for group 4 (free supporters) was below 0.5 suggesting a low stability of this cluster. However, we decided to keep these four clusters because of their meaningful interpretability. This and the within cluster homogeneity were our most important criteria to choose the number of clusters as presented here.

Despite these limitations, our cluster analysis identified four groups of farmers and meaningful differences in their attitudes. Although the bias regarding the proportions of the groups is unratable, a relevant impact on the interpretation of the groups is not presumed.

## Conclusions

Significant differences among the attitudes of farmers regarding paratuberculosis control were identified, ranging from supporters with or without knowing their herd status regarding paratuberculosis to deniers. A group that we named ‘informed sceptics’ did not consider paratuberculosis a dangerous epizootic, but would enrol in a control programme if their herd was affected by paratuberculosis. In contrast to the deniers, these farmers were identified as relevant candidates for future enrolment. Across all groups, the most important motivations for enrolment were protecting future profitability and improving animal health. Lack of accuracy of diagnostic tests and their costs were mentioned most frequently by the deniers as obstacles and should be addressed in communication regarding paratuberculosis control. Because of the relevant differences between the four groups of farmers, tailored communication strategies for the specific target groups are required to enhance participation in paratuberculosis control in a voluntary setting.

## Methods

### Eligible interviewees

Two German federal states with long-lasting voluntary paratuberculosis control programmes, Thuringia and Saxony, were selected for the present study. Both programmes were similar in their design and focused on herd specific counseling of herd managers to improve farm hygiene, testing of all cows in the herd annually using faecal culture, culling of shedders as quickly as possible, risk-based purchase of cattle, and certification of herds as non-suspect regarding paratuberculosis [16]. Herd level true prevalence was similar in both regions and was estimated to be 56% (95% confidence interval [CI] 42–70%) for Thuringia and 34% (95% CI: 20–48%) for Saxony [22]. An anonymous survey was performed in both regions during 2013 and 2015. Roughly 625 questionnaires were distributed among cattle farmers together with boot-swab sampling sets for a free and anonymous herd-level paratuberculosis testing.

### Boot-swab sampling and testing for MAP genome

The sampling sets were equipped with boot-swabs for MAP detection as previously described [19, 20]. Sampling was performed by the farmers in accordance with detailed instructions. They were advised to ship the sample together with the questionnaire as quickly as possible after sampling to the laboratory of the Thuringian Animal Health Service. Samples were stored at − 20 °C until testing for MAP genome by qPCR. The VetMAX™ MAP real-time PCR Screening Kit (Life Technologies, Darmstadt, Germany) was used for samples collected between 2013 and 2014, and on the samples collected in 2015 the ADIAVET™ PARATBREAL TIME Kit (Biomerieux, Nürtingen, Germany) was applied as described previously [20, 21]. Sensitivity and specificity of both PCR test applied on boot swab were similar [21].

### Questionnaire

Based on expert opinion, a questionnaire was developed by a group of four well-experienced veterinarians of the Animal Health Services of Thuringia and Saxony, all with long-lasting experience in paratuberculosis control. The questionnaire was evaluated by each of them during at least two on-farm visits in face-to-face interviews with farmers. The final questionnaire consisted of 27 questions and was subdivided into four parts:

1. demographics of the respondents,
2. attitude, opinions and level of awareness regarding paratuberculosis,
3. motivations for enrolment in a paratuberculosis control programme, and
4. obstacles that hamper participation.

Part 1 comprised the following questions and possible answers (in brackets):

- Indicate your postcode (first three numerals).
- Indicate your type of production (dairy, beef, mixed, fattening)
- Indicate the size of your cattle herd (≤10, 11–100, 101–500, > 500)
- Do you already participate in a voluntary control programme (yes, no)?
- When did you first learn about paratuberculosis? (today, this year, ≤5 years ago, > 5 years ago).

In part 2–4, questions had to be answered using a symmetric scale with either “yes”, “rather yes”, “rather no” or “no”. Questions are indicated in Table 3 and in Figs. 1, and 2, 3.

### Data analysis

All analyses were applied at the level of the respondent. First, the distributions of the categorised herd sizes (Table 1) and of the federal states (Saxony, Thuringia) among the participants were compared to the respective distributions of the eligible interviewees to evaluate the representativeness of the sample. Second, a cluster analysis was performed to identify groups of farmers with similar attitudes towards paratuberculosis and its control. Finally, for every question of the questionnaire, independence of the distribution of the answers from the identified clusters was tested.

To evaluate the representativeness of the sample, a hierarchical Bayesian Poisson regression model for three-dimensional contingency tables was fitted:

$${\mathrm{y}}_{\mathrm{i}}\sim \mathrm{Pois}\left({\uplambda}_{\mathrm{i}}\right)$$

with y_i_ being the count of cell *i* and

$${\uplambda}_{\mathrm{i}}=\exp \left({\upalpha}_0+{\upalpha}_1{\mathrm{p}}_{\mathrm{i}}+{\upalpha}_2{\mathrm{f}}_{\mathrm{i}}+{\upalpha}_3{\mathrm{s}}_{\mathrm{i}}+{\upalpha}_{12}{\mathrm{p}}_{\mathrm{i}}{\mathrm{f}}_{\mathrm{i}}+{\upalpha}_{13}{\mathrm{p}}_{\mathrm{i}}{\mathrm{s}}_{\mathrm{i}}\right)$$

where *p*, *f* and *s* represented participation, federal state, and herd size class, respectively. The sample would be treated as representative if the counts per federal state and per herd size class were pairwise independent from participation, i.e. if the 95% highest posterior density intervals of the respective interaction parameters (*α*_*12*_ and *α*_*13*_) covered the null. Independent normally distributed priors with mean 0 were used for the regression parameters (*α*_*•*_). The variance of the prior distribution for the intercept was 10^6^. For the other coefficients, the variances of the prior distributions were chosen from folded *t*-distributions with mean 0, scale 0.001 and 2 degrees of freedom [31]. The posterior distributions of the model parameters were estimated using Markov chain Monte Carlo and Gibbs sampling. The sampling was performed in JAGS version 4.2.0 [32] via R version 3.3.1 [33] and the additional package runjags version 2.0.4–2 [34]. Three sampling chains with different initial values were run for 7.5 * 10^5^ steps after a burn-in period of 7.5 * 10^3^ steps. After thinning, 7.5 * 10^3^ samples remained in each chain. Convergence was assessed using trace plots and the Brooks-Gelman-Rubin diagnostic.

A hierarchical cluster analysis was performed to identify groups of farmers with similar attitudes towards paratuberculosis and its control. Only the eight questions of part 2 were included in this analysis. The answers to questions of parts 3 and 4 were interpreted separately from part 2 to avoid disaccords. As the possible answers were on an ordinal scale, their ranks were used. A missing answer was treated as neutral answer in the middle of the rank scale. The dissimilarity matrix was calculated based on Gower’s distance metric. The cluster agglomeration was performed using Ward’s minimum variance method. The number of meaningful clusters was determined by employing the following criteria [35]:

1. the interpretability of the attitude of the farmers within the cluster facilitated by the visualisation in heatmaps,
2. the within cluster homogeneity, assessed via the average distance within clusters,
3. the between cluster separation, expressed as separation index [35], and
4. the cluster stability, assessed by the Jaccard similarity value calculated as mean from nonparametric bootstrap resampling [36].

The cluster analysis was conducted in R version 3.3.1 [33] using the additional packages cluster version 2.0.4 [37] and fpc version 2.1–10 [38].

The differences between the identified clusters were investigated by comparing the frequencies of the answers per question. Therefore, for every question, a Bayesian Poisson regression model for two-dimensional contingency tables was fitted:

$${\mathrm{y}}_{\mathrm{i}}\sim \mathrm{Pois}\left({\uplambda}_{\mathrm{i}}\right)$$

with y_i_ being the count of cell *i* and

$${\uplambda}_{\mathrm{i}}=\exp \left({\upalpha}_0+{\upalpha}_1{\mathrm{c}}_{\mathrm{i}}+{\upalpha}_2{\mathrm{a}}_{\mathrm{i}}+{\upalpha}_{12}{\mathrm{c}}_{\mathrm{i}}{\mathrm{a}}_{\mathrm{i}}\right)$$

where *c* and *a* represented the cluster and the answer class, respectively. If the 95% highest posterior density interval of at least one interaction parameter (*α*_*12*_) excluded the null, the frequencies of the answers to the question would not be considered as independent from the clusters. The estimation of the posterior distributions was performed as described for the analysis of the representativeness except for the lengths of the sampling chains. In the present analysis, 3 * 10^3^ steps were used as burn-in and afterwards the chains were run for another 1.5 * 10^5^ steps. After thinning, 3 * 10^3^ samples remained in each chain. Convergence was assessed using trace plots and the Brooks-Gelman-Rubin diagnostic.

## Supplementary Information

**Additional file 1.** csv Individual answers to the questionnaire, results of the boot-swab samples and identified clusters as table of comma separated values.

## Data Availability

All data generated or analysed during this study are included in this published article and its additional files.
